# Supplementary figures and images for: Hyponatremia and Cancer: From Bedside to Benchside
Source: Cancers (Basel). 2023 Feb 13;15(4):1197. doi: 10.3390/cancers15041197 (PMC9953859; doi:10.3390/cancers15041197)

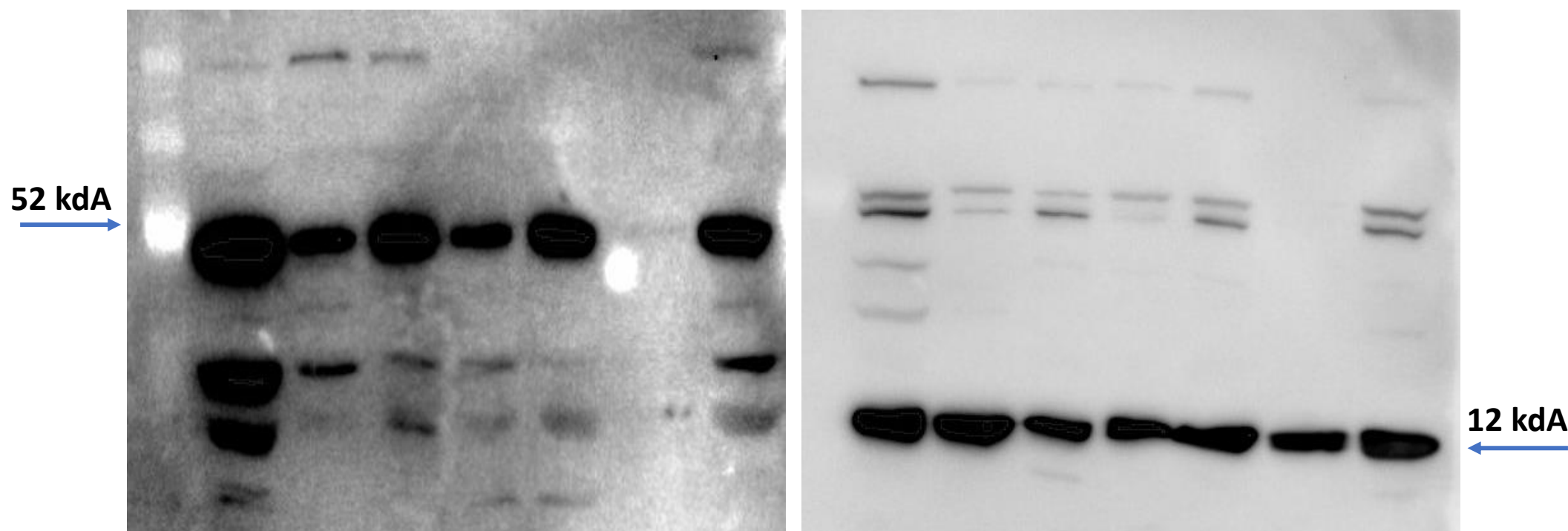

**Figure S1.** Whole gels for AVPR2 (left) and  $\beta$ 2-microglobulin (right).

Supplement: Supplementary file 1 [file cancers-15-01197-s001.zip › cancers-2162923-supplementary.pdf]
